# Clinical Care Delivery in Chest Pain Patients Without an Acute Coronary Syndrome—A Retrospective Cohort Study

**DOI:** 10.3390/jcm14041372

**Published:** 2025-02-19

**Authors:** Anas Alrefaee, Sherif Eltawansy, Abbas Alshami, Paweł Łajczak, Ndausung Udongwo, George Ayob, Jeffrey Selan

**Affiliations:** 1 Division of Cardiology, Jersey Shore University Medical Center, Neptune, NJ 07753, USA; anas.alrefaee@hmhn.org (A.A.); abbas.alshami@hmhn.org (A.A.); jeffrey.selan@hmhn.org (J.S.); 2 Department of Medicine, Jersey Shore University Medical Center, Neptune, NJ 07753, USA; ndausung.udongwo@hmhn.org; 3 Department of Biophysics, Faculty of Medical Sciences in Zabrze, Medical University of Silesia, 40-043 Katowice, Poland; 4 Hackensack-Meridian School of Medicine, Seton Hall University, Nutley, NJ 07601, USA; george.ayob@hmhn.org

**Keywords:** chest pain, heart Function tests, clinical observation units, emergency room visits, acute coronary syndrome

## Abstract

**Background:** Chest pain is a prevalent and critical complaint among patients in emergency departments (EDs) across the United States. Professional societies have refined clinical guidelines to establish the most effective diagnostic pathways for identifying obstructive coronary artery disease. However, many healthcare systems do not adhere to the guideline-validated clinical pathways and instead order repeat diagnostic testing. This study evaluated the efficiency of care delivered to chest pain patients in our tertiary medical center. **Methods:** We performed a retrospective chart review of patients presenting to our ED with acute chest pain between November and December 2022, collecting information about chest pain, the testing received, and their outcomes. The data were then reviewed to determine clinical practice patterns. **Results:** We included 342 patients, with a mean age of 54 years; 54.7% of study participants were females. Patients who were eventually admitted from the ED (46.5%, *n* = 159) were either under observation or inpatient status. Furthermore, 16.6%, *n* = 57, of patients had an ischemic evaluation within the preceding year. Physicians documented a HEART score in 24.6%, *n* = 84 of the patients. While HEART score is a considerable factor utilized by admitting physicians to triage incoming patients, 39%, *n* = 62, of all admitted patients had a low HEART score (<3) and a negative ischemic evaluation within the past year. **Conclusions:** This single-center retrospective analysis of care delivery for non-ACS (acute coronary syndrome) chest pain patients demonstrated that the HEART score was not thoroughly documented in the study population. This resulted in an overperformance of inpatient ischemic testing, with an increased length of stay and costs for the institution and healthcare system. This study serves as a quality improvement initiative to explore similar data within their institutions and as a reminder of the importance of utilizing validated clinical pathways to streamline clinical care and reduce healthcare costs.

## 1. Introduction

Chest pain is a significant reason for emergency department (ED) visits, as it is the second leading cause in the US [1]. Acute coronary syndrome (ACS) is typically ruled in or out through serial cardiac enzymes and electrocardiograms [2]. Once ACS has been ruled out, patients are typically risk-stratified into low-, intermediate-, and high-risk categories that might progress to ACS. Multiple studies have proven the accuracy of risk assessment scoring systems [3]. Healthcare system policymakers introduced hospital chest pain observation units to admit patients within the intermediate- and high-risk categories for an ischemic evaluation [4].

As chest pain is one of the most common reasons patients seek medical attention, the American College of Cardiology established guidelines for evaluating acute or stable chest pain in the outpatient and emergency rooms. Such guidelines are established to expedite assessing and managing patients exhibiting clinically significant chest pain. These guidelines consider CAD risk factors and prior ischemic testing (functional or anatomic) with clinical and objective testing tools to minimize unnecessary testing in low-risk populations and identify those most likely to benefit from additional testing [5]. The HEART score is one scoring pathway for risk stratifying patients presenting to the emergency room. Six-week major adverse cardiac events (MACE) occurred in 1.7% of low-score patients (0–3), 16.6% of intermediate scores (4–6), and 50.1% of high scores (7–10) [3]. Other diagnostic modalities include the TIMI score (Thrombolysis in Myocardial Infarction) and the HFA/CSANZ (Heart Foundation of Australia and Cardiac Society of Australia and New Zealand) rule [6]. Since chest pain is such a common presenting complaint to the ED, we would expect a high cost on the national level. Yet, it is one of the reasons for the presentation to the ED with the lowest overall mortality rate [7]. Inpatient admissions declined from ED visits, with a decline in aggregate admission costs and an increase in mean admission costs. That means there is a trend of reducing admission of chest pain cases to the hospital despite the increased visits to US EDs nationwide. A study that implemented a national database found that there were 42.5 million ED visits for chest pain in the US from 2006 to 2016. Furthermore, visit rates per 100,000 persons increased by 37% between 2006 and 2016 [8]. Providers utilized different approaches to manage chest pain observation cases. American College of Cardiology/American Heart Association (ACC/AHA) released guidelines in 2021 that guide evaluating this population of patients. ACC/AHA patient-centric algorithms for acute chest pain incorporate history, physical exam, and troponin levels for risk stratification to direct testing choices [5]. The advancement of diagnostic methods for various coronary diseases, including ACS, provides more accuracy in identifying underlying coronary artery disease, even with atypical presentations. However, that led to problems stemming from excessive test ordering with inappropriate resource utilization. One study estimated an annual cost of USD 501 million and 491 future cases of cancer attributed to inappropriate testing [9]. Julie C. Will et al. reported a doubling in the rates of stress testing during the periods from 1999 to 2002 and 2003 to 2006 [10]. The rates remain high despite campaigns by professional societies such as Choosing Wisely to reduce over-testing. Between 2005 and 2012, Kini V et al. reported that using other modalities offset the 14.9% decrease in nuclear single-photon emission computed tomography [11]. Applying the HEART score added more guidance; however, it might not be utilized perfectly by healthcare providers given its evident subjectivity and inconsistency between physicians who rated patients while describing chest pain quality from history [12].

Healthcare system stakeholders are evaluating ED chest pain observation units’ performance to assess clinical safety and cost efficiency. Continuing efforts are to utilize these units under the updated guidelines to manage chest pain cases effectively. More patients might benefit from these observation units, which would reduce costs and length of stay [7]. ED physicians should consider prior testing and integrate it with the patient’s risk profile to decide how necessary it is to repeat ischemic testing [13]. There is a recommendation to use EKG stress testing as the first line for those patients. However, there is excessive utilization of radionuclide myocardial perfusion imaging, which adds more financial burden on the healthcare system and more radiation exposure to patients. Researchers noted that teaching hospitals use radionuclide myocardial perfusion imaging less often than non-teaching hospitals [14]. Physicians are applying HEART scores on a broader level to help stratify patient risks; a low HEART score provides high sensitivity and negative predictive value for predicting major adverse cardiac events [15].

Our study aimed to investigate the course of patients in a tertiary care facility with an urban teaching setting who presented with chest pain that did not prove to be an evident ACS from the first presentation. We analyzed the number of patients, the testing they received, and their disposition. We offered to review the impact of the HEART score as one of the most commonly used scoring systems in clinical decision-making pathways. We intended to evaluate the performance of this patient category management and performance effectiveness towards better care delivery in a significant clinical presentation to the ED.

## 2. Methods

Setting: The study location was a tertiary referral center with a 691-bed capacity, designated as a university-level academic center in central New Jersey.

Participants: This retrospective study included adult patients aged 18 years or above who presented to a tertiary care facility in New Jersey, USA ED, with the chief complaint of chest pain. We retrospectively reviewed charts of patients who visited the ED between November 2022 and December 2022.

Our study did not include detailed criteria of EKG; on the other hand, according to exclusion criteria, all patients did not have EKG changes that would indicate clear evidence of acute myocardial infarction, such as ST elevation, or new left bundle branch block.

**Figure 1:**
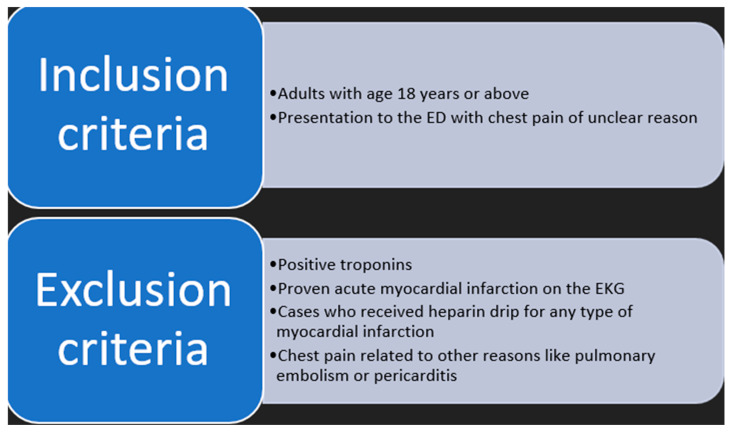
Criteria for inclusion and exclusion of participants.

We excluded patients whose presentation was consistent with ACS, including those with positive troponins and an ACS diagnosis based on electrocardiogram (EKG) criteria (Figure 1).

High-Sensitivity Troponin I (TnIH) is processed through the Siemens Atellica^®^ IM-10997840 (100 tests) 10997841 (500 tests), 40 Liberty Boulevard, Malvern, PA 19355, USA.

No CK-MB was carried out as it is not part of the current practice in the hospital; work up in the ED included blood work like blood count, chemistry, and high sensitivity troponin. That was in addition to chest X-ray and CT scan of the chest as warranted. If the ED physician decided to proceed further, the case would be referred to the floor hospitalist physician for admission to a possible cardiology consultation. Afterwards, the patient would be placed in an observation unit with subsequent ischemic evaluation.

### 2.1. Outcomes

The primary outcome was quantifying the utilization of the HEART score risk stratification tool in patients admitted for chest pain evaluation.

The secondary outcome included identifying the diagnostic testing used to evaluate those patients and the outcomes of those tests.

### 2.2. Study Design

We retrieved the following patient data, including date of presentation, age, gender, and past medical history: hypertension (HTN), hyperlipidemia (HLD), smoking history, obesity based on BMI more than 30 kg/m^2^, history of coronary artery disease (CAD), history of myocardial infarction (MI), history of peripheral artery disease (PAD), history of cerebrovascular disease (CVA), history of transient ischemic attack (TIA), chronic kidney disease (CKD), end-stage renal disease (ESRD), and if the patient is on dialysis or not (HD).

We reviewed the past surgical history for coronary artery bypass graft (CABG), history of percutaneous coronary intervention (PCI), or heart transplant.

We reviewed family history for premature CAD (male parent or sibling < 55 years old, female < 65 years old) and if there was a sudden cardiac death (SCD).

We verified if the patient had undergone any ischemic evaluation before the index presentation. The type of ischemic evaluation, whether within a year from the presentation or older, and the test results were all identified. The types of ischemic evaluation included stress testing or left heart catheter (LHC). If performed, we inquired about the timing of the patient’s coronary computed tomography angiogram (CCTA) and whether it was conducted within a year, between one and two years, or over two years before the presentation.

**Figure 2:**
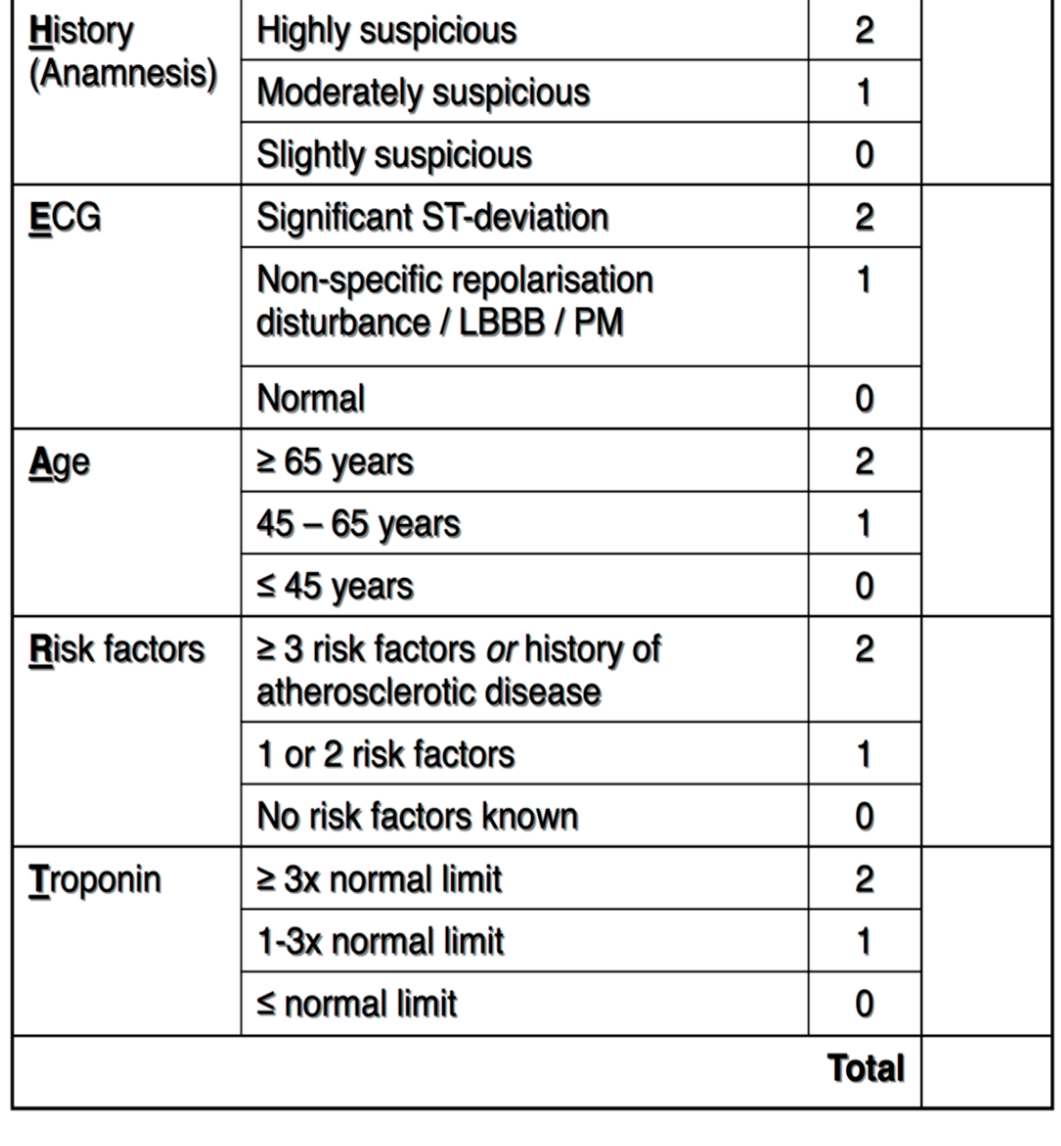
HEART score description.

The patients were risk-stratified before undergoing any testing using the HEART score (Figure 2). The HEART score was independently calculated regardless of whether it was documented in the chart. High-sensitivity troponin (HS-cTn) was used in the patients’ blood work. 

We evaluated the chart for diagnostic testing performed while the patient was admitted. Testing included any stress testing, LHC, or CCTA. The outcome of any diagnostic testing and all therapeutic interventions were recorded. Disposition, mortality, and length of stay were also evaluated.

Patients who had one positive and one negative ischemic test were not considered to have a negative ischemic evaluation. We reported a negative LHC if it was normal or non-obstructive and negative stress when it was normal. A mildly abnormal stress test was considered positive.

This study was conducted under the Strengthening the Reporting of Observational Studies in Epidemiology (STROBE) guidelines [16]. We adhered to the observational cohort guideline.

Our organization’s institutional review board (IRB) reviewed the study protocol. Due to the study’s retrospective nature, it provided patients’ consent and Health Insurance Portability and Accountability Act (HIPAA) waivers (Study ID: Pro2022-0347). This study followed Good Clinical Practice guidelines and the Declaration of Helsinki [16].

### 2.3. Variables and Data Sources

Clinical information was obtained from patients’ electronic medical records (EMRs) in the Epic system (Epic Systems Corporation, Verona, WI, USA). Physicians collected and validated all data through a manual chart review. Data collected included patient demographics, comorbidities, cardiovascular risk factors, physical examination findings, home medications, imaging findings, EKGs, and telemetry strips.

### 2.4. Sample Size Calculation

We intended to include all the patients admitted to our institution during the study period.

### 2.5. Statistical Analysis

The Shapiro–Wilk test and histograms were used to examine the normality of continuous variables. Categorical variables were described as frequency and percentages. We described continuous variables as means with standard deviations or medians with interquartile ranges (IQRs), as appropriate. We used the chi-square test to compare categorical variables and the Mann–Whitney-U test to compare continuous non-parametric variables. We used the intraclass correlation coefficient (ICC) with a two-way mixed model for absolute agreement to assess the consistency between the reported heart scores and those independently calculated. We performed all analyses using IBM SPSS statistics version 25.0 (IBM Corporation, Armonk, NY, USA). We used an alpha value (*p*) of 0.05 to ascertain statistical significance

## 3. Results

A total of 342 patients were included in this retrospective analysis. Eleven patients had a negative ischemic evaluation within one year; five patients were admitted (under observation or inpatient status). A prior ischemic evaluation was documented in 40.9% of patients (*n* = 140), including 16.6% (*n* = 57) of patients with ischemic testing performed within the past year. The median age of participants was 54 years (IQR 42–65), with 54.7% being females (*n* = 187). The baseline characteristics of patients are displayed in Table 1. The most common forms of prior ischemic evaluation were a stress test (18.4% of the total included sample, *n* = 63) and LHC (18.4%, *n* = 63), followed by coronary CTA (4.1%, *n* = 14).

### 3.1. Utilization of Heart Score in Disposition

**Figure 3:**
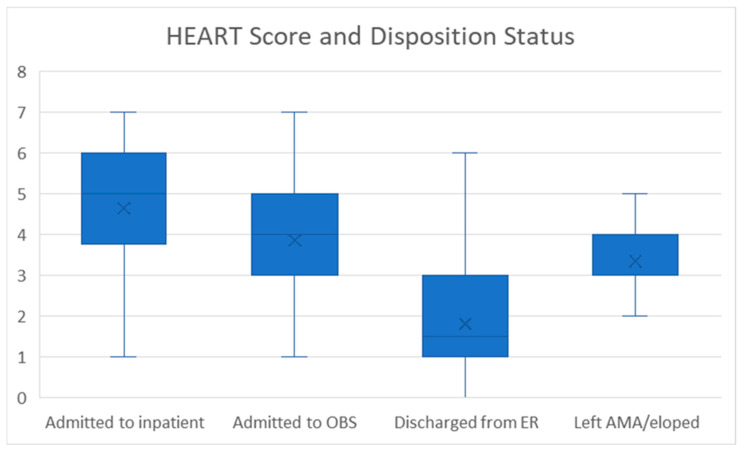
HEART score (for all patients) and disposition status. Results are presented as median, IQR, and range.

Of the 342 patients, 49.1% (*n* = 168) were discharged home from the ED, 46.5% (*n* = 159) were admitted to the hospital (under observation or inpatient status), and 4.4% (*n* = 15) left the hospital against medical advice. Physicians documented a HEART score in 24.6% of the patients (*n* = 84), and all included patients’ charts were used to calculate the HEART scores independently. For the 84 patients with reported HEART scores, there was a good agreement between the reported and the calculated scores (ICC 0.879, 95% CI 0.813–0.921). The calculated HEART score was higher in patients admitted to the hospital than those who were discharged from the ED [4 (IQR 3–5) versus 1.5 (IQR 1–3)]. Chart review demonstrated that 35.8% (*n* = 57) of admitted patients had a HEART score of 3 or less, with 62 patients (40% of all admitted patients) being admitted despite having both a low HEART score (<3) or a negative ischemic evaluation within the preceding year. Figure 3 shows the analysis of HEART score and disposition status among all 342 patients.

**Figure 4:**
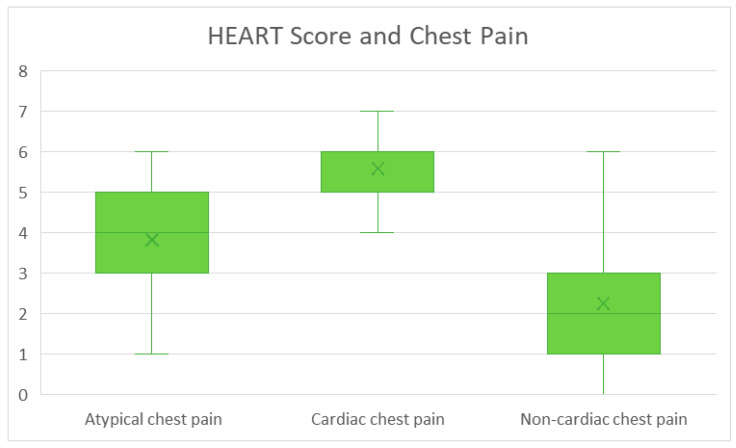
HEART score (for all patients) and chest pain type. Results are presented as median, IQR, and range.

**Figure 5:**
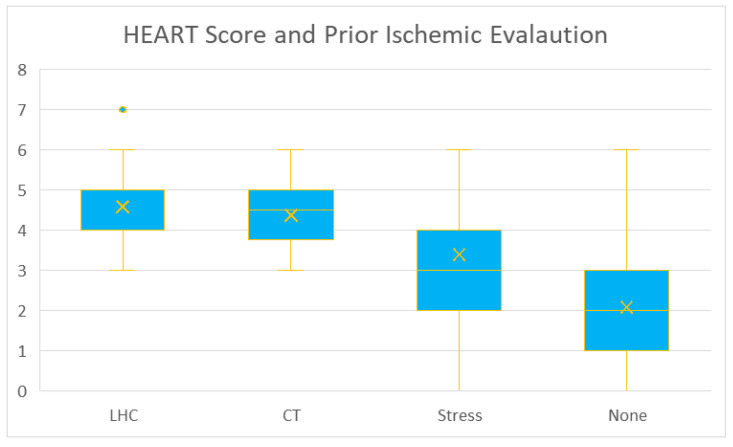
HEART score (for all patients) and prior ischemic evaluation. Results are presented as median, IQR, and range.

**Figure 6:**
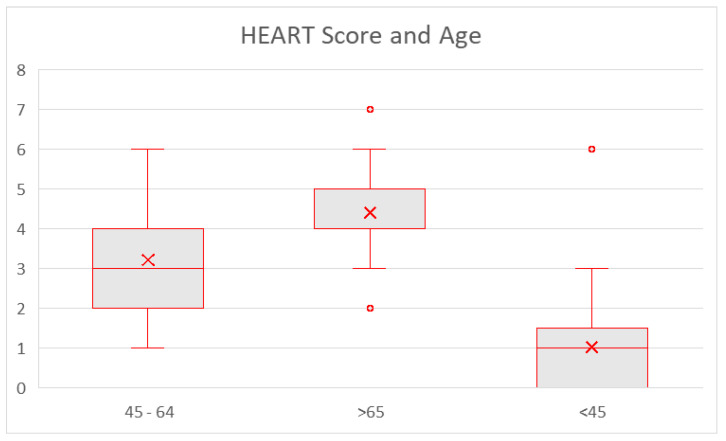
HEART score (for all patients) and age group. Results are presented as median, IQR, and range.

We performed an analysis with the chi-squared test on the observed relationship between baseline demographic details and calculated HEART score. We found statistically significant results (*p* < 0.001) in insurance type, hypertension, hyperlipidemia, diabetes, coronary artery disease, prior MI, peripheral artery disease, coronary artery bypass graft surgery, percutaneous coronary intervention, hospital stay, disposition type, cardiology consultation, history of cardiac chest pain (Figure 4), prior ischemic evaluation (Figure 5), the patient age (lowest scores in <45 years subgroup—Figure 6), and obesity, (*p* = 0.027).

We found no statistically significant differences in gender (*p* = 0.243), smoking (*p* = 0.073), chronic kidney disease (*p* = 0.153), end-stage renal disease (*p* = 0.136), stress test results (*p* = 0.143), or LHC results (*p* = 0.22). 

### 3.2. Inpatient Workup

**Figure 7:**
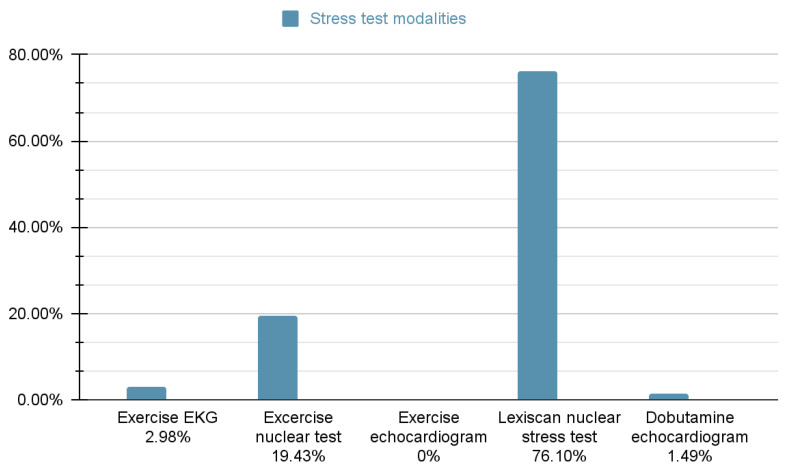
Percentages of stress tests performed.

Of the admitted patients, 51.57% (*n* = 82) went on to have an inpatient ischemic workup during that admission. This group was stratified into testing modules, with stress testing as the most common form of inpatient workup (69.5%, *n* = 57), followed by LHC (20.7%, *n* = 17), then CCTA (9.75%, *n* = 8). Stress testing included different modalities (Figure 7).

Of the patients with inpatient workups, 31.7% (*n* = 26) had a HEART score of 3 or less, and 15.9% (*n* = 13) had an ischemic evaluation within the preceding year. Only one patient with an inpatient ischemic workup had a positive stress test.

In LHC tests, there were four patients with normal results, two patients with non-obstructive CAD, six patients with obstructive who received stents, and five patients with obstructive CAD who did not receive stents.

In CCTA, there were five patients with normal outcomes, three patients with non-obstructive CAD, and no patients with obstructive CAD.

### 3.3. Safety Data

Two patients underwent inpatient CABG, and six patients underwent inpatient PCI. Of the patients discharged home from the ER, only 6% (*n* = 10) were discharged with a plan for outpatient stress testing, and no patient had a referral to a cardiologist documented in their charts.

### 3.4. Cost Data

In total, 75% of patients had a length of stay of at least one day. The hospital cost for 24 h of observation is approximately USD 9600.

## 4. Discussion

Our study included patients with significant comorbidities like hypertension, hyperlipidemia, diabetes, and smoking. More than 40% of our study sample patients had a prior ischemic evaluation, and more than 16% had an ischemic review performed within 1 year of admission. The study’s results also showed that the utilization of heart score was only performed in 24.6% of patients, and 35.8% of patients were admitted with a documented low-risk HEART score (<3). Of the patients admitted, more than half had an ischemic workup in the inpatient setting, and the majority had resulted in negative non-invasive workup during their admission.

The observed associations between the HEART score and various demographic and clinical factors provide further insights into its clinical utility. The lower HEART scores in younger patients (<45 years) align with the generally lower prevalence of cardiovascular disease in this population, highlighting the importance of considering age in score interpretation. The significant associations with established cardiovascular risk factors, such as hypertension, hyperlipidemia, diabetes, and obesity, as well as prior cardiac procedures (CABG/PCI), are consistent with existing knowledge and reinforce the score’s ability to reflect underlying cardiovascular risk. The relationships between the HEART score and hospital stay/disposition, as well as cardiology consultation, likely reflect the score’s predictive value: patients with higher scores are more likely to be admitted, have longer stays, and require specialist input. The significant association with a history of cardiac chest pain further validates the HEART score’s sensitivity to this crucial presenting symptom. Conversely, the lack of significant associations with smoking status, chronic kidney disease, end-stage renal disease, stress test results, and LHC results warrants further consideration. The non-significant finding for smoking, for instance, may be attributable to the relatively small sample size or limitations in the accuracy of smoking status documentation within the electronic health record. Similarly, the absence of a significant association with CKD/ESRD, stress test results, and LHC results could be due to data limitations or other unmeasured confounding factors, rather than a true lack of association. Further investigation is needed to explore these relationships in greater detail

HEART scores could be a great asset to healthcare providers; previous studies showed that adopting the HEART score could reduce hospital admissions by 15% without leading to unpredicted cardiovascular events [17]. A study yielded a strong correlation between the documented HEART score and patient cardiovascular outcome, with 2.5% of the patient sample with a HEART score of 0–3 points requiring a CABG 11 days after admission, 20.3% of HEART score of 4–6 points had an endpoint, and a score of 7–10 points led to 72.7% with a positive outcome [18].

HEART score has drawbacks despite being a practical method that would direct practitioners. A study demonstrated that the HEART score has 0.5% missing odds of patients that might end up with a major adverse cardiovascular event, which might add hesitancy to providers in solely adopting it, eventually leading to underutilization [19]. Inconsistency in calculating the HEART score might add fear, pressuring physicians to comfortably rely on it. While there are continuous variables like age and troponin, other factors like chest pain quality and risk factors assessment might not be stable [18]. Emergency department (ED) physicians might not accept the fact-driven data exhibiting that patients with HEART scores (HS) ≤3 have 30-day Major Adverse Cardiovascular Effect (MACE) rates of 0.6% to 3.6% for the fear of medicolegal consequences [20].

This yields a significant area of improvement for healthcare facilities, including ours, where a closer evaluation of AHA/ACC guidelines can be performed and practiced for achieving more cost-effective care. With 75% of patients included in this study having an average length of stay of at least 1 day, the resulting cost accrued for admission was more than USD 1,500,000 for two months at our hospital. Continued improvement in implementing the protocol-driven risk stratification for chest pain admissions may result in tremendous healthcare cost reductions, as evidenced by our single-center experience during this two-month study period.

In the end, different factors affect the ED physician, hospitalist, or cardiologist’s decision to admit or discharge patients presenting with chest pain, such as the reliability of cardiological follow-up. Our study has no clear documentation of how the ED referred the patient to a follow-up visit, including time and place. Only 6% of patients in our study had clear documentation of a scheduled plan for outpatient stress testing if they did not do it in the hospital. Although documentation might not be accurate here and might have missed many follow-up details, it helps improve communication and compliance between the patient and the healthcare system. ED physicians are responsible for ensuring patient safety upon discharge, which may result in significant inpatient over-testing [21,22,23,24].

Researchers spend ongoing efforts trying to discover more innovative gadgets physicians could utilize to precisely diagnose chest pain with actual underlying cardiac diseases most feasibly and practically. Among different tools available, measuring high-sensitivity troponins, utilizing a CT scan of the chest with an angiogram to rule out pulmonary embolism or aortic dissection as a cause of chest pain, applying clinical pathways of acute chest pain (including HEART score), lung ultrasound [25], and point-of-care ultrasound [26].

### Study Limitations

There are some limitations to this study. As this is a retrospective observational study, the data collected are limited to that available via electronic medical records. Decisions made regarding patient-centered care that may not have been documented or accessible would not have been considered within this study’s scope. Moreover, the sample included in our study was of limited size and in a chronological timeline, which may not represent the population admitted to our institution.

## 5. Conclusions

This single-center, retrospective analysis of care delivery for non-ACS (acute coronary syndrome) chest pain patients demonstrates the infrequent utilization of validated chest pain scoring tools for risk stratification and the over-reliance on inpatient ischemic testing. This results in increased length of stay and costs for the institution and healthcare system. Furthermore, ischemic testing is often repeated despite recent testing within the past year. We also found that inpatient non-invasive ischemic testing in non-ACS chest pain patients is typically negative and thus would be unlikely to change their clinical course. This study serves not only as a quality improvement initiative within our institution but as a recommendation for others to explore similar data within their institutions and as a reminder of the importance of utilizing validated clinical pathways to streamline clinical care and reduce healthcare costs.

## Figures and Tables

**Table 1:** Summary of the baseline characteristics of the study participants.

Variable	Median (IQR) or Frequency (%)
Age (years)	54 (42–65)
Gender, female	187 (54.7)
Insurance	
Medicare	31 (9.1)
Medicaid	13 (3.8)
Private	133 (38.9)
Other	165 (48.2)
Hypertension	178 (52)
Hyperlipidemia	142 (41.5)
Diabetes Mellitus	43 (12.6)
Smoking	85 (24.9)
Obesity (BMI ≥ 30)	92 (26.9)
Known history of CAD	64 (18.7)
Prior myocardial infarction	27 (7.9)
Peripheral arterial disease	6 (1.8)
History of CVA or TIA	21 (6.1)
Chronic kidney disease	8 (2.3)
End-stage renal disease	2 (0.6)
Prior coronary artery bypass graft surgery	11 (3.2)
Prior percutaneous coronary intervention	50 (14.6)

## Data Availability

Data is contained within the article supplementary material. The original contributions presented in this study are included in the article/supplementary material (Supplementary Table S1). Further inquiries can be directed to the corresponding author.

## Supplementary Materials

The following supporting information can be downloaded at: https://www.mdpi.com/article/10.3390/jcm14041372/s1.

## Abbreviations

ED	Emergency department
ACS	Acute coronary syndrome
ACC	American College of Cardiology
AHA	American Heart Association
HTN	Hypertension
HLD	Hyperlipidemia
EKG	Electrocardiogram
CAD	Coronary artery disease
MI	Myocardial infarction
PAD	Peripheral artery disease
CVA	Cerebrovascular disease
TIA	Transient ischemic attack
CKD	Chronic kidney disease
ESRD	End-stage renal disease
HD	Hemodialysis
LHC	Left heart catheter
CCTA	Cardiac computed tomography angiogram
CABG	Coronary artery bypass graft
PCI	Percutaneous coronary intervention
SCD	Sudden cardiac death
STROBE	Strengthening the Reporting of Observational Studies in Epidemiology
IRB	Institutional review board
IQR	Interquartile ranges
ICC	Intraclass correlation coefficient
HIPAA	Health Insurance Portability and Accountability Act
TIMI	Thrombolysis in Myocardial Infarction
HFA/CSANZ	Heart Foundation of Australia and Cardiac Society of Australia and New Zealand
